# Development and long‐term stability of a comprehensive daily QA program for a modern pencil beam scanning (PBS) proton therapy delivery system

**DOI:** 10.1002/acm2.12556

**Published:** 2019-03-28

**Authors:** Suresh Rana, Jaafar Bennouna, E. James Jebaseelan Samuel, Alonso N. Gutierrez

**Affiliations:** ^1^ Department of Radiation Oncology Miami Cancer Institute Baptist Health South Florida Miami FL USA; ^2^ Department of Radiation Oncology Herbert Wertheim College of Medicine Florida International University Miami FL USA; ^3^ Department of Physics School of Advanced Sciences VIT University Vellore India

**Keywords:** daily QA, pencil beam scanning, proton therapy

## Abstract

**Purpose:**

The main purpose of this study is to demonstrate the clinical implementation of a comprehensive pencil beam scanning (PBS) daily quality assurance (QA) program involving a number of novel QA devices including the Sphinx/Lynx/parallel‐plate (PPC05) ion chamber and HexaCheck/multiple imaging modality isocentricity (MIMI) imaging phantoms. Additionally, the study highlights the importance of testing the connectivity among oncology information system (OIS), beam delivery/imaging systems, and patient position system at a proton center with multi‐vendor equipment and software.

**Methods:**

For dosimetry, a daily QA plan with spot map of four different energies (106, 145, 172, and 221 MeV) is delivered on the delivery system through the OIS. The delivery assesses the dose output, field homogeneity, beam coincidence, beam energy, width, distal‐fall‐off (DFO), and spot characteristics — for example, position, size, and skewness. As a part of mechanical and imaging QA, a treatment plan with the MIMI phantom serving as the patient is transferred from OIS to imaging system. The HexaCheck/MIMI phantoms are used to assess daily laser accuracy, imaging isocenter accuracy, image registration accuracy, and six‐dimensional (6D) positional correction accuracy for the kV imaging system and robotic couch.

**Results:**

The daily QA results presented herein are based on 202 daily sets of measurements over a period of 10 months. Total time to perform daily QA tasks at our center is under 30 min. The relative difference (Δ_rel_) of daily measurements with respect to baseline was within ± 1% for field homogeneity, ±0.5 mm for range, width and DFO, ±1 mm for spots positions, ±10% for in‐air spot sigma, ±0.5 spot skewness, and ±1 mm for beam coincidence (except 1 case: Δ_rel_ = 1.3 mm). The average Δ_rel_ in dose output was −0.2% (range: −1.1% to 1.5%). For 6D IGRT QA, the average absolute difference (Δ_abs_) was ≤0.6 ± 0.4 mm for translational and ≤0.5° for rotational shifts.

**Conclusion:**

The use of novel QA devices such as the Sphinx in conjunction with the Lynx, PPC05 ion chamber, HexaCheck/MIMI phantoms, and myQA software was shown to provide a comprehensive and efficient method for performing daily QA of a number of system parameters for a modern proton PBS‐dedicated treatment delivery unit.

## 1 INTRODUCTION

The number of proton therapy centers in the US continues to grow as there is an increasing interest in the use of protons to treat cancer patients.^1^ Currently, proton centers employ different beam delivery techniques such as double scattering (DS), uniform scanning (US), and/or pencil beam scanning (PBS). In the last few years, a number of existing proton centers have upgraded their beam delivery technique from DS/US to PBS. The majority of new proton centers is now configured with a more advanced PBS beam delivery technique that has been shown to deliver a more conformal dose when compared to DS/US techniques.^2^ However, PBS proton beam delivery has uncertainties associated with its spot size and spatial position. Such demand for advanced PBS delivery warrants a comprehensive understanding and monitoring of PBS beam characteristics. In addition to advances in proton beam delivery, image guidance used for proton treatments has also evolved in recent years. In the past, the primary imaging modality in proton centers had been planar kV x‐ray technique. Newer proton centers are incorporating imaging modalities such as cone‐beam computed tomography (CBCT) and surface imaging (SGRT). Due to the increase in delivery complexity of PBS and the use of multi‐modality image‐guidance systems for patient treatments, there is a need to establish a comprehensive daily quality assurance (QA) program that assesses safety, mechanical, dosimetric, and imaging parameters to ensure safe radiation delivery — similar to the recommendations set forth by AAPM TG‐142 for photon‐based delivery systems. Additionally, certain proton centers may employ multi‐vendor hardware and software for daily patient treatment. For these centers, interconnectivity becomes a critical element to assess and testing of data transfer among different softwares (e.g., beam delivery, imaging, record, verify systems, etc.) should be integrated as part of the daily QA program.

Several authors have published on proton daily QA using either commercial or in‐house developed devices.^3^, ^4^, ^5^, ^6^, ^7^, ^8^, ^9^ Arjomandy et al.^3^ published a paper in 2009 providing an overview of QA procedures implemented at The University of Texas M. D. Anderson Proton Therapy Center at Houston (PTC‐H). Arjomandy et al.^3^ verified the output, distal range, and spread‐out Bragg Peak (SOBP) for daily QA of DS proton beams using the solid‐water plastic. In 2012, Ding et al.^4^ initially investigated the use of Sun Nuclear Daily‐QA 3 (DQA‐3) device (Sun Nuclear Inc., Melbourne, FL, USA) for daily QA of US proton beams. In 2014, Lambert et al.^5^ extended the use of DQA‐3 for daily QA of PBS proton beams. For PBS dosimetric tests, Lambert et al.^5^ evaluated the output, range, spot sigma, and position. Since the DQA‐3 was originally designed for photon and electron daily QA, authors^4^, ^5^ manufactured an in‐house phantom to use with the DQA‐3. Actis et al.^6^ published on PBS daily QA in 2017 utilizing an in‐house developed phantom that can accommodate multi‐leaf ionization chamber (MLIC). Actis et al.^6^ included beam characteristics (spot width, size, and position), range, and dose output for the dosimetric component of daily QA. Another PBS daily QA paper was published in 2017 by Bizzocchi et al.^7^ and investigated the use of MatriXX‐PT (IBA Dosimetry, Schwarzenbruck, Germany) with an in‐house phantom to evaluate range, spot size and position, and dose output. In a more recent paper, Younkin et al.^8^ utilized the DQA‐3 along with an in‐house developed phantom to evaluate dose output, beam range, and spot position as part of PBS daily QA.

The above‐mentioned studies^4^, ^5^, ^6^, ^7^, ^8^ demonstrate that investigators have used in‐house developed phantoms and software in conjunction with commercially available devices for PBS daily QA. Moreover, the detectors used in previous PBS Daily QA studies^4^, ^5^, ^6^, ^7^, ^8^ were limited to DQA‐3, MLIC, and MatriXX‐PT. Recently, a novel PBS dedicated commercial device Sphinx (IBA Dosimetry, Schwarzenbruck, Germany) has been made available to proton therapy centers. In order to be able to quantify specific PBS beam characteristics, the Sphinx must be used in conjunction with the Lynx (IBA Dosimetry, Schwarzenbruck, Germany) and a parallel‐plate (PPC05) ionization chamber (IBA Dosimetry, Schwarzenbruck, Germany). Given the novelty of the device and no published literature on the experience of the Sphinx and Lynx for PBS daily QA, this work focuses on our clinical implementation and long‐term results when incorporating these devices for the dosimetric component of our PBS daily QA. In addition to evaluating the dosimetric component of PBS proton beams, this work highlights the importance of a comprehensive daily QA program and addresses the need to develop other components such as safety, mechanical, and imaging tests to ensure safe radiotherapy treatment deliveries. Daily QA tests presented in this work may serve beneficial to proton centers looking to develop and implement a comprehensive daily QA program based on recently developed commercially available detectors and phantoms.

## 2 MATERIALS AND METHODS

**Figure 1:**
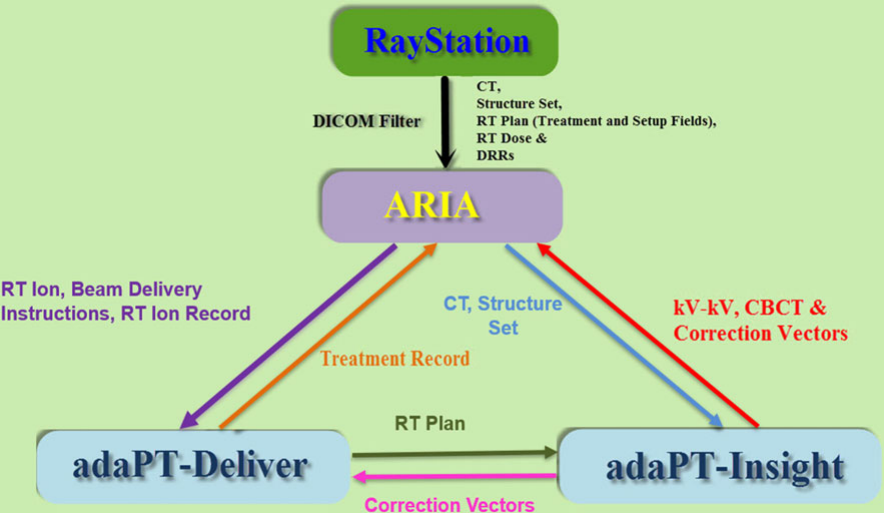
Flow chart of data transfer among RayStation, ARIA, adaPT‐Deliver (beam delivery), and adaPT‐Insight (imaging) in a ProteusPLUS pencil beam scanning proton therapy system.

Our proton center is structured as a multi‐vendor hardware and software platform environment. PBS proton plans are generated in RayStation (v.6.1.1.2; RaySearch Laboratories, Stockholm, Sweden), whereas ARIA (v.13.7; Varian Medical Systems, Palo Alto, CA) is used as the department record and verify system. IBA (Ion Beam Applications, Louvain‐la‐Neuve, Belgium) provides the ProteusPLUS PBS proton therapy system, which includes adaPT‐Deliver (v.11.0.3) for beam delivery and adaPT‐Insight (v.2.1.0d) for imaging (kV‐kV x ray and kV‐CBCT). Additionally, the CatalystPT (C‐RAD, Uppsala, Sweden) system is used for surface imaging and gating applications. The flow chart of data transfer among the various software entities is presented in Fig. 1.

### 2.A Beam delivery system (BDS)

**Figure 2:**
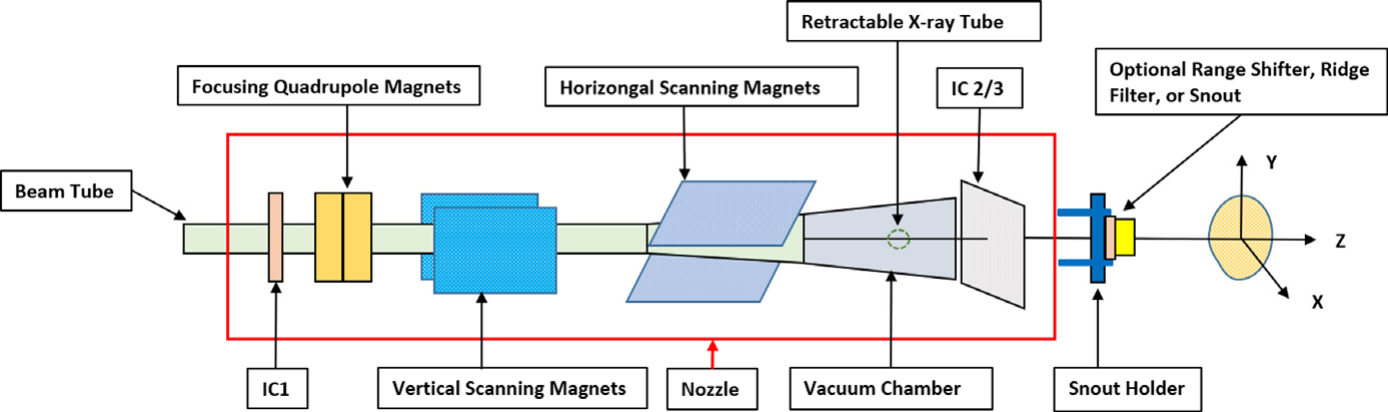
Schematic representation of the beam delivery system equipment in the pencil beam scanning (PBS) treatment mode for an IBA ProteusPLUS gantry‐based system. Note: The x‐ray tube (portal) is located in the PBS dedicated nozzle pre‐assembly, which is under vacuum. The x‐ray tube is retracted from the beam line during proton beam irradiation.

A PBS proton beam is delivered using a PBS dedicated nozzle (Fig. 2). As the proton beam enters the nozzle, an ionization chamber 1 (IC1) verifies the alignment of the beam at the nozzle entrance. A set of two focusing quadrupole magnets focus the proton beam at the isocenter. The proton beam is then scanned in Y direction by a vertical scanning magnet followed by scanning in X direction with a horizontal scanning magnet. In order to direct the beam to a particular location on a target, the beam position is steered using magnetic fields. Ionization chambers 2 and 3 (IC2/3) monitor beam characteristics real‐time (beam size, position, and flatness) and dose just before the proton beam exists the nozzle. Snout holder allows the movement of accessary drawer, which can include an optional range shifter (pre‐absorber) and snout. At our center, a range shifter of 7.5 cm water equivalent thickness is used for clinical cases as necessary.

### 2.B Imaging systems

The kV x‐ray imaging system includes two gantry mounted, x‐ray tubes that rotate with the gantry. The first x‐ray tube (portal) is located in the PBS dedicated nozzle pre‐assembly, which is under vacuum. The x‐ray tube is retracted from the beam line during the proton beam irradiation. The flat panel detector of portal (SAD = 119.4 cm, SID = 177.0 cm, active pixel area = 28.2 cm × 40.6 cm, and active pixel resolution: 2232 × 3200 pixels) is located in front of the nozzle. The second x‐ray tube (orthogonal) is fixed to one of the gantry structural beams. The x‐ray beam axis is perpendicular to the proton beam axis and to the gantry rotation axis. The orthogonal tube in conjunction with its flat panel detector (SAD = 264.2 cm, SID = 317.1 cm, active pixel area = 43 cm × 43 cm, and active pixel resolution: 2874 × 2840 pixels) is used for the kV‐CBCT acquisition. In addition to the x ray based imaging system, the CatalystPT, a three‐camera surface imaging system, is used to setup patients prior to x ray based imaging, monitor patient position and posture during treatment, and enable beam gating. The three cameras are positioned to maximize field coverage with the outer cameras being 43° from the center camera.

### 2.C Record and verify system

ARIA (v13.7) receives computed tomography (CT) images, DICOM structure set, RT Plan, RT Dose, and DRR images from RayStation. ARIA also receives the treatment record from adaPT Deliver and images (kV planar/CBCT images) from adaPT Insight.

### 2.D Phantoms and detectors

**Figure 3:**
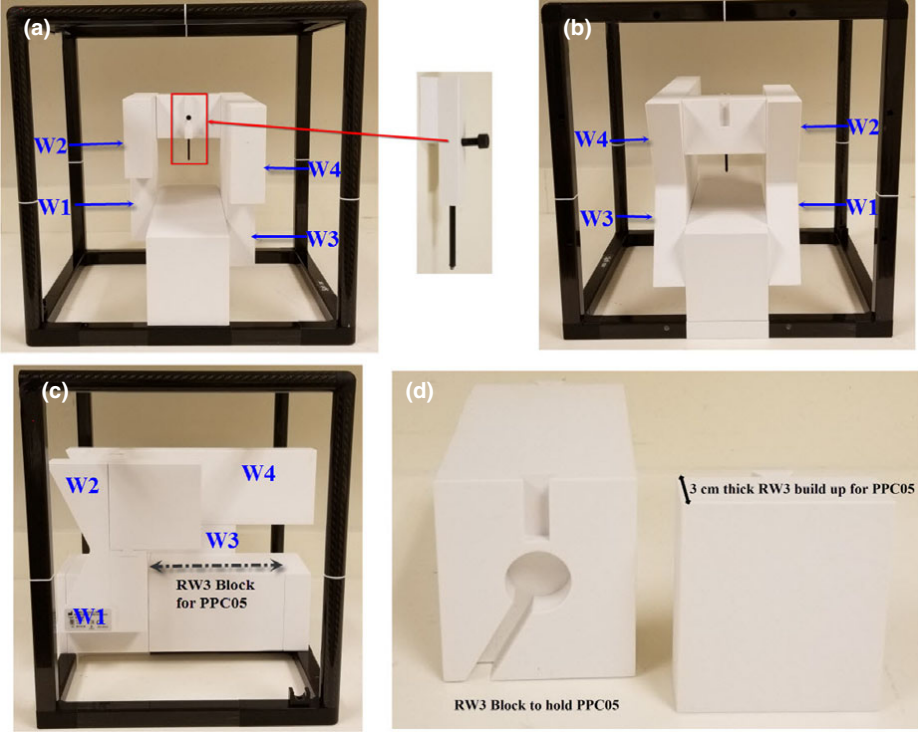
*(a)* The Sphinx device is shown with the fiducial insert for x‐ray vs proton beam coincidence. (a‐c) The RW3 blocks with wedges (W1, W2, W3, and W4) are shown in frontal (a), back (b), and side (c) views. The W1, W2, W3, and W4 are used to measure the ranges, width, and distal‐fall‐off of energies 106, 145, 172, and 221 MeV, respectively. (d) The RW3 block is shown with the cutout for the PPC05 parallel plate chamber as well as the 3 cm thickness buildup that is placed in front of the chamber.

The Sphinx phantom has a carbon frame with dimension of 540 mm × 400 mm × 400 mm (Fig. 3). The carbon frame contains the markers for verification of laser alignment. The phantom incorporates four wedges with various thicknesses for verifying the constancy of different proton beam energies (106, 145, 172, and 221 MeV). The fixed solid water block (RW3) and insert RW3 have mass density of 1.045 g/cm^3^ and electron density of 3.386 × 10^23^/cm^3^. The RW3 insert has dimensions of 35 mm width, 100 mm height, and variable length (250, 200, and 100 mm).

The four wedges are utilized to calculate the energy related parameters such as range, width, and distal‐fall‐off (DFO). The energy calculation algorithm^10^ implemented within myQA software, version 2017‐002 (2.9.23.0) calculates the slight signal generated by the radiation delivered over the RW3 wedge. The first derivative of this rising part of signal is then calculated in order to identify the physical edge of the corresponding RW3 block.^10^ The final depth‐dose curve is calculated by assigning a value of depth to each pixel of the image.^10^ The values of depths are extrapolated from data interpolated with a cubic spline fit.^10^ For better understanding on the range calculation using wedge, readers are advised to refer to work published by Shen et al.^11^ and Deng et al.^12^

The phantom also has an insert containing a pin with a fiducial at its tip which is placed at the isocenter (Fig. 3). A dedicated RW3 insert (160 mm × 90 mm × 100 mm) contains a notch for a PPC05 chamber for dose output constancy check. The PPC05 is covered with 3 cm thickness RW3 block so the chamber has a 3 cm build up (Fig. 3). The PPC05 chamber can then be connected to an electrometer for dose output measurement. The phantom setup allows the in‐air measurement of spots at the level of the Lynx. The Lynx is a gadolinium‐based scintillation detector (active surface area = 300 mm × 300 mm) with a pixel resolution of 0.5 mm. A detailed description of the Lynx is provided by Russo et al.^13^

For imaging quality assurance, the multiple imaging modality isocentricity (MIMI) phantom along with the HexaCheck phantom (Standard Imaging, Middleton, WI, USA) are used to perform daily, six‐dimensional (6D) image‐guided radiation therapy (IGRT) QA of the IBA adaPT‐Insight software and LEONI (LEONI Healthcare, Chartres France) robotic couch. The HexaCheck acts as a base for the MIMI phantom and allows for the introduction of a fixed 2.5° mechanical displacement in the yaw, pitch, and roll directions. For more information on the clinical use of MIMI and HexaCheck, readers are advised to refer to white paper.^14^

### 2.E Workflow

Our current daily QA workflow includes two daily QA plans based on two sets of devices: (a) Sphinx, Lynx, and PPC05 and (b) MIMI and HexaCheck.

#### 2.E.1 Sphinx, Lynx, and PPC05

**Figure 4:**
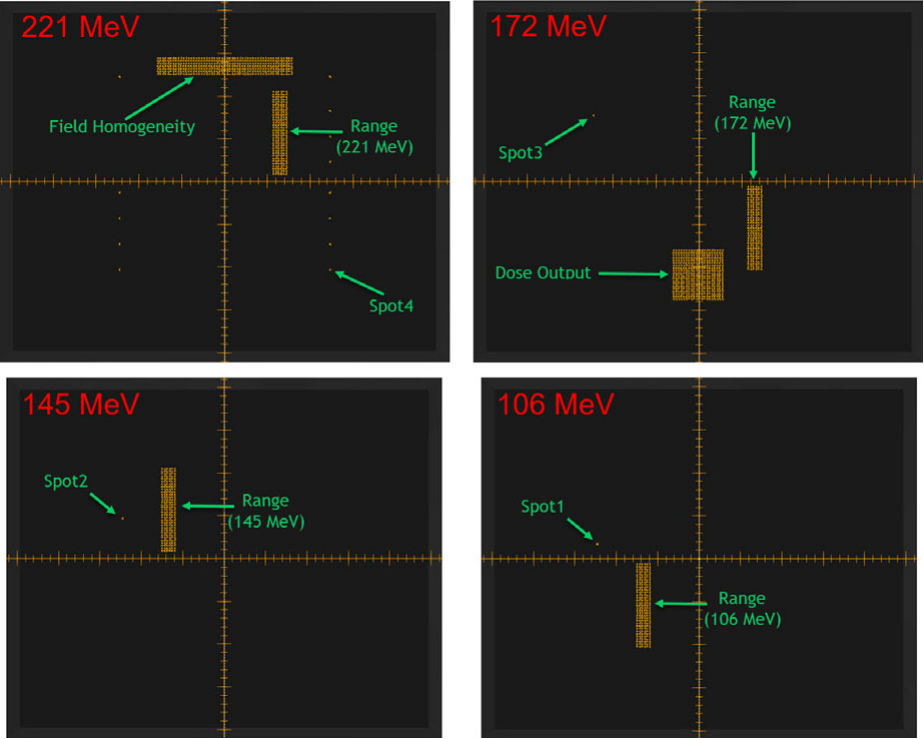
The spot map of a plan created in RayStation for four different proton beam energies (221, 172, 145, and 106 MeV) for the dosimetric testing of the pencil beam scanning daily quality assurance using the Sphinx and Lynx devices is shown.

**Figure 5:**
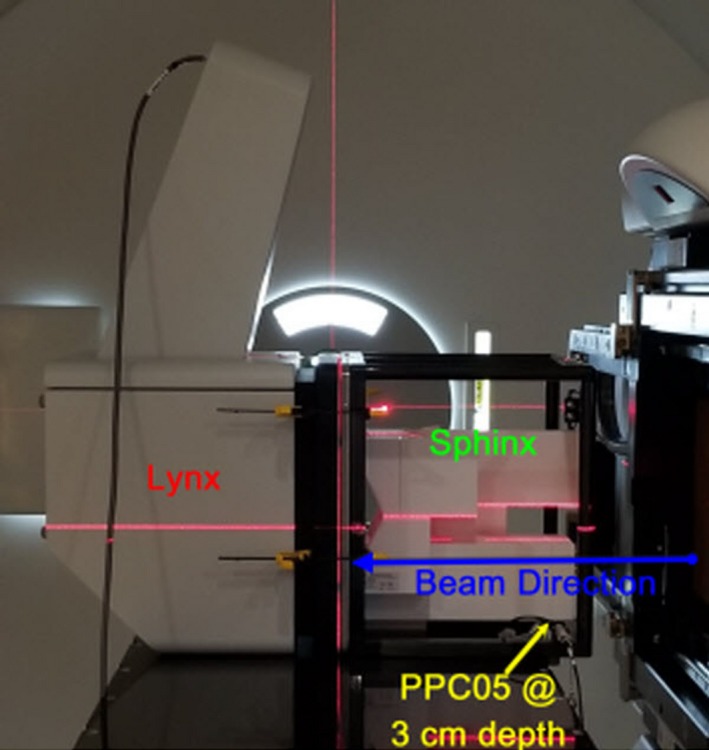
The daily couch top setup of the Sphinx, Lynx, and PPC05 for pencil beam scanning dosimetric testing of the daily quality assurance procedure is shown. The gantry is set at 90° with the robotic couch being set to 0°.

**Figure 6:**
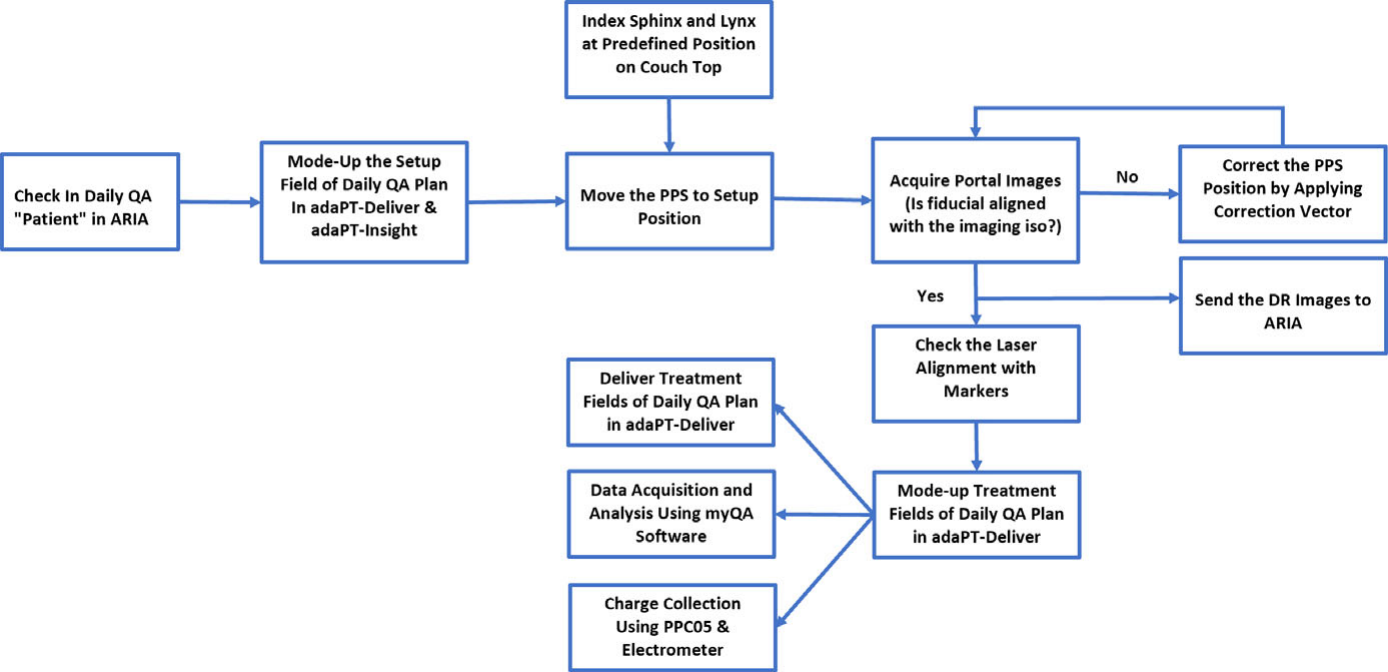
The workflow for the dosimetric component of the daily quality assurance of a pencil beam scanning treatment unit using the Sphinx, Lynx, and PPC05 is shown.

A daily QA plan was generated in RayStation (v.6.1.1.2) with spot map of four different energies (Fig. 4). In order to mimic patient treatment, a daily QA plan is delivered using adaPT‐Deliver on ProteusPLUS proton therapy system through ARIA. Dosimetry measurements are performed using a single couch top setup with the Sphinx, Lynx, and PPC05 chamber (Fig. 5) For PBS daily QA dosimetric quantification, tests (Table 1) are categorized into: (a) spot position, size, and skewness, (b) distal and proximal range, width, and DFO, (c) radiation and imaging coincidence, (d) field homogeneity, and (e) dose output. For evaluation and analysis, myQA software (IBA Dosimetry, Schwarzenbruck, Germany) was utilized for tests #a–d and an in‐house excel sheet and DOSE^2^ electrometer (IBA Dosimetry, Schwarzenbruck, Germany) were used for test #e. Additionally, verification of patient positioning system (PPS) displacement and lasers alignment is accomplished with the same setup. The workflow using Sphinx, Lynx, and PPC05 is presented in Fig. 6. The total time for this workflow is from 15 to 20 min without system interruptions.

**Table 1:** Overview of daily quality assurance tests for a proton pencil beam scanning delivery system

	Test items
Safety	Door interlock (beam off)
	Audio/visual monitor(s)
	Intercom
	Beam on indicator
	Alarm indicator
	X‐ray on indicator
	Room radiation monitor
	Room search/clear button
	Beam pause
	Beam stop
	Beam delivery controller reset
	Collisional interlocks
Mechanical, imaging, and OIS connectivity	Laser localization
	Imaging and treatment coordinate coincidence
	Positioning/repositioning (translational & rotational)
	Gantry angle
	Range shifter detection
	Dual source kV‐kV x‐ray image acquisition
	CBCT acquisition
	Connectivity between OIS and delivery unit software
	X‐ray vs surface imaging isocenter coincidence
Dosimetry	Spot position
	Spot sigma
	Spot skewness
	Distal range
	Proximal range
	Width
	Distal‐fall‐off (DFO)
	Imaging vs proton beam isocenter coincidence
	Field homogeneity
	Dose output

#### 2.E.2 MIMI and HexaCheck

**Figure 7:**
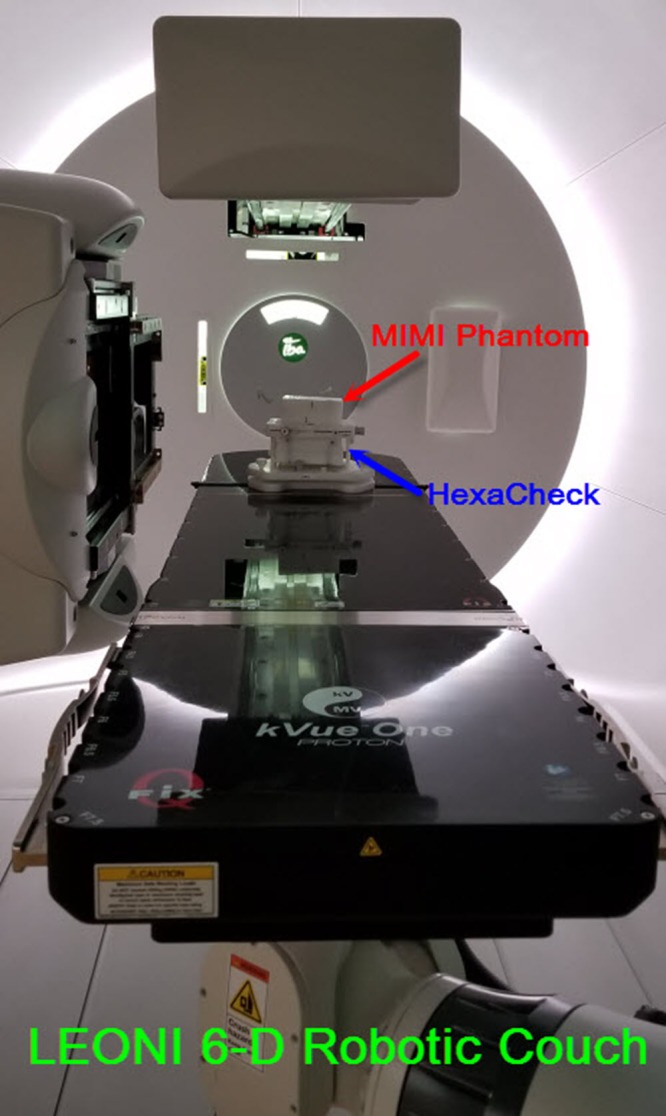
The couch top setup of the MIMI and HexaCheck phantoms at the predefined position of the LEONI six‐dimensional robotic couch. The MIMI phantom is positioned with both translational and rotational offsets applied as shown above.

**Figure 8:**
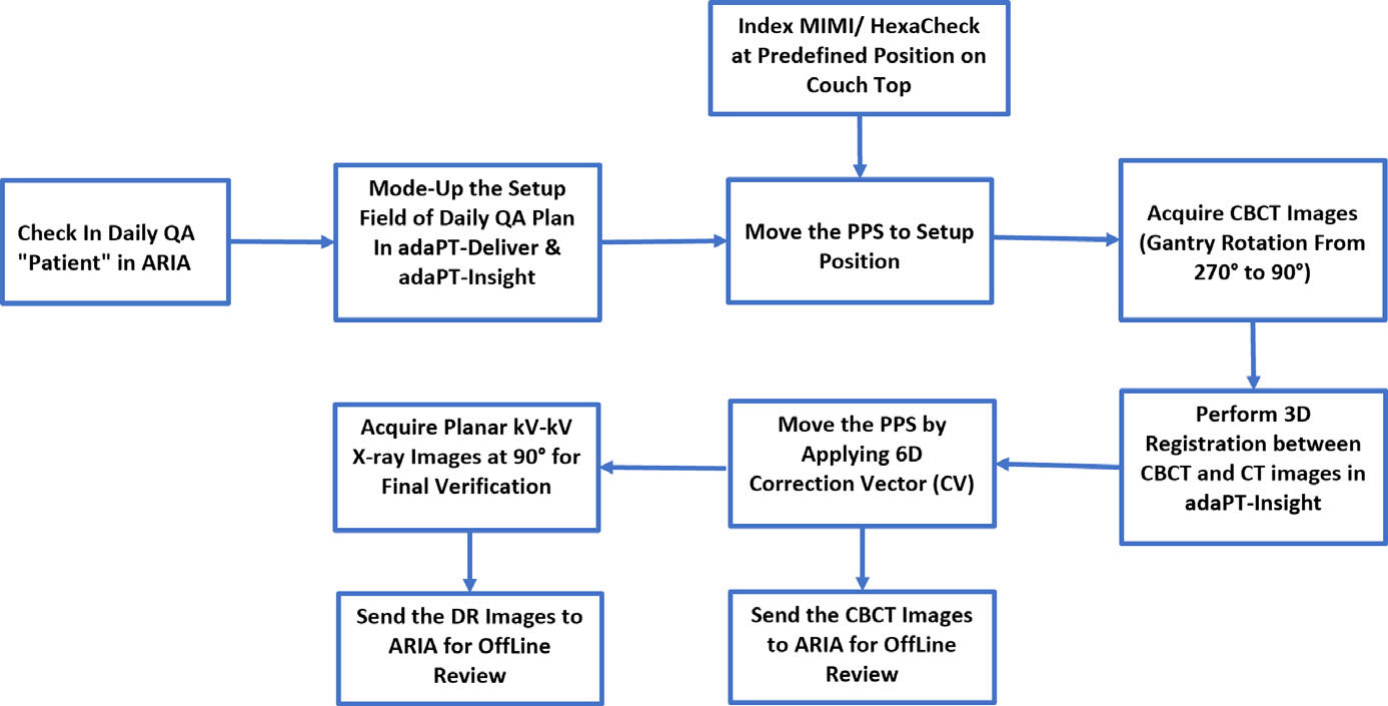
The workflow for the imaging component of the daily quality assurance of a pencil beam scanning treatment unit using the MIMI and HexaCheck is illustrated.

A treatment plan with kV‐kV and CBCT setup fields was generated in RayStation using the CT images of the MIMI phantom. The plan treatment isocenter was defined at the center of the MIMI. The MIMI is placed in the HexaCheck and indexed to the couch top such that MIMI is aligned to the known translational and rotational offset shifts (Fig. 7) Specifically, the known translational shifts were −13.4 mm in the lateral, −9.1 mm in the longitudinal, and 10.8 mm in the vertical directions, whereas the known rotational shifts were 2.2° for the pitch, −2.2° for the roll, and 3.5° for the yaw. First, a CBCT is acquired with gantry rotation from 270° to 90°, and the acquired CBCT images are registered to the reference CT images of the MIMI in adaPT‐Insight to obtain the 6D correction vector. The difference between the daily correction vectors (translational and rotational) and baseline values are calculated using Eq. (1) provided below. After applying correction vector to the PPS, kV‐kV x‐ray imaging is performed to verify the final position of the MIMI phantom is accurate. Both the kV‐kV x‐ray and CBCT images are transferred to ARIA for offline review. The workflow using MIMI and HexaCheck is presented in Fig. 8. The total time for this workflow is about 10 min.

(1) Δ(p)=M(p) ‐ B(p)

where, p = translational (e.g., lateral) or rotational parameter (e.g., yaw); M(p) = daily measured value of parameter, p; B(p) = baseline value of parameter, p; Δ(p) = difference between measured and baseline values of parameter, p.

## 3 RESULTS

**Figure 9:**
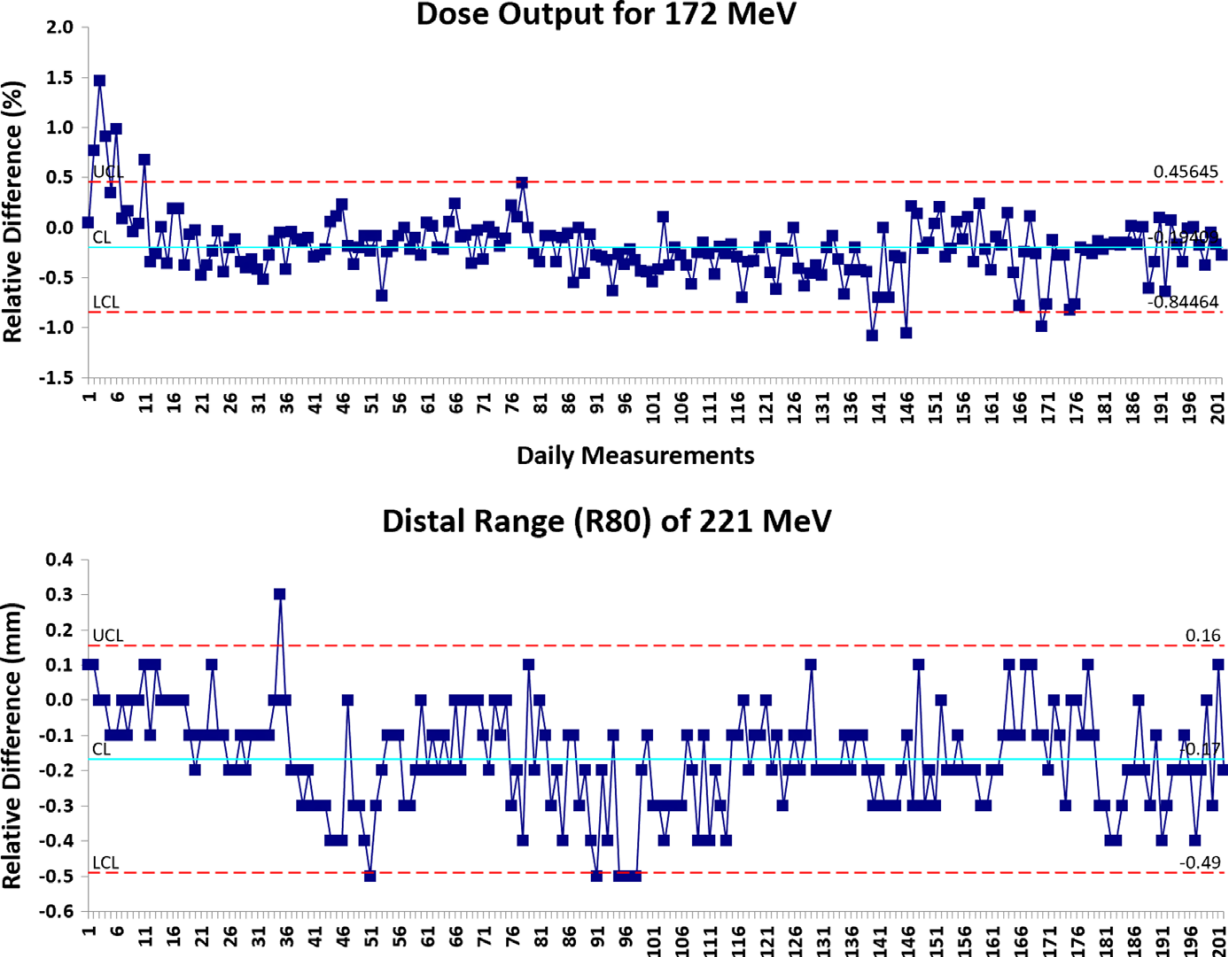
The XbarR control charts for the dose output and distal range (R80) are shown. Statistical process control methods were used to determine the stability of the beam parameters. Upper control limit (UCL) = +3*σ* and lower control limit (LCL) = −3*σ* were used to determine if individual measurements required action.

The daily QA results presented herein are based on a set of 202 daily measurements over the period of 10 months on an IBA gantry‐based ProteusPLUS PBS proton therapy system. The analysis of results was carried out in two steps. First, the relative difference (Δ) was calculated by comparing daily (D) measurements against baseline (B) measurements. Second, a statistical process control (SPC) analysis was performed to assess the temporal stability of each parameter and determine whether the various parameters of the system were in statistical control. The QI Macros (KnowWare International, Denver, CO) add‐on statistical analysis package (v.2018) for Microsoft Excel was used for the statistical analysis. Specifically, for the Δ of each evaluated parameter, the upper control limit (UCL), lower control limit (LCL), and average values were calculated using a XbarR control chart in QI Macros. An example of the control chart for the dose output of the 172 MeV beam as well as the distal range (R80) of the 221 MeV beam is displayed in Fig. 9. The UCL and LCL are defined by +3*σ* and −3*σ*, respectively, from the average value. The 3*σ* means 99.73% of the values lie within three standard deviations of the mean.

### 3.A Dose output and field homogeneity

**Figure 10:**
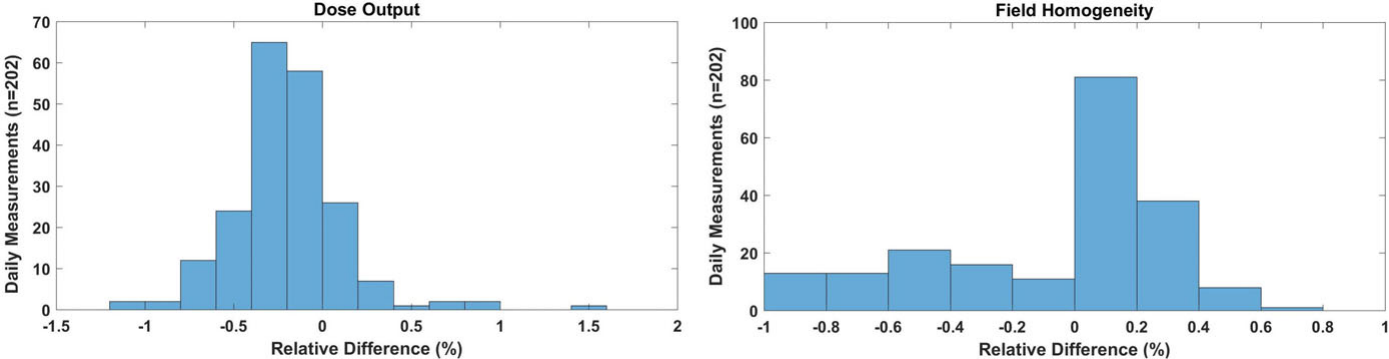
(Left) Daily dose output for 172 MeV pencil beam scanning (PBS) proton beam;(Right) Daily field homogeneity for 221 MeV PBS proton beam. [The relative difference (Δ) was calculated by comparing daily (D) measurements (*n* = 202) against baseline (B) measurement.]

Dose output and field homogeneity were evaluated for energies of 172 and 221 MeV, respectively. Table 2 and Fig. 10 show that the average Δ in dose output was −0.2% (range, −1.1%–1.5%) relative to baseline, and the Δ in field homogeneity was within ±1% (range, −1.0%–0.7%). The 3*σ* of the dose output and field homogeneity were ±0.7% and ±0.3%, respectively (Table 2).

**Table 2:** Results of dose output, field homogeneity, range, width, distal‐fall‐off (DFO), and x‐ray vs proton beam coincidence based on daily QA measurements (*n* = 202)

	Energy (MeV)	Avg.	Range	SPC		
				3*σ*	UCL	LCL
Dose output (%)	E172	−0.2	−1.1–1.5	±0.7	0.5	−0.8
Field homogeneity (%)	E221	0.0	−1.0–0.7	±0.5	0.5	−0.6
R80‐distal range (mm)	E106	−0.1	−0.3–0.1	±0.3	0.2	−0.3
	E145	−0.2	−0.3–0.1	±0.3	0.1	−0.4
	E172	0.2	0.0–0.5	±0.3	0.5	−0.1
	E221	−0.2	−0.5–0.3	±0.3	0.2	−0.5
R80‐proximal range (mm)	E106	−0.1	−0.3–0.1	±0.3	0.2	−0.4
	E145	−0.2	−0.4–0.1	±0.3	0.1	−0.5
	E172	0.1	−0.3–0.4	±0.3	0.4	−0.3
	E221	−0.2	−0.5–0.5	±0.3	0.1	−0.6
Width (mm)	E106	0.0	0.0–0.1	±0.2	0.2	−0.1
	E145	0.0	−0.1–0.2	±0.2	0.2	−0.1
	E172	0.2	0.0–0.4	±0.2	0.4	−0.1
	E221	0.0	−0.2–0.3	±0.2	0.2	−0.2
Distal‐fall‐off (mm)	E106	0.0	−0.1–0.1	0	0.0	0.0
	E145	0.0	0.0–0.1	±0.1	0.2	−0.1
	E172	0.0	0.0 – 0.1	±0.1	0.1	0.0
	E221	0.0	−0.1–0.1	±0.1	0.1	−0.1
Beam coincidence‐X (mm)	E106	0.4	−0.8–1.3	±0.7	1.1	−0.4
Beam coincidence‐Y (mm)	E106	0.1	−0.7–0.9	±0.5	0.6	−0.4

A relative difference (Δ) was calculated by comparing daily (D) measurements against baseline (B) measurements. Upper control limit (UCL) and lower control limit (LCL) are based on statics process control (SPC) charts. UCL = +3*σ* and LCL = ‐3*σ* are from the average value.

### 3.B Energy, width, and DFO

The energy, width, and DFO were evaluated for 106, 145, 172, and 221 MeV energies. Table 2 shows the Δ in distal and proximal ranges (R80) were within ±0.5 mm for all four energies. For both the distal and proximal ranges, the 3*σ* (Table 2) of all four energies was ±0.3 mm. For both the width and DFO, the Δ was within ±0.4 mm. The 3*σ* (Table 2) of width for all four energies was ±0.2 mm, whereas the 3*σ* of DFO was ±0.1 mm for energies 145, 172, and 221 MeV and 0 mm for 106 MeV.

### 3.C Spots characteristics

**Figure 11:**
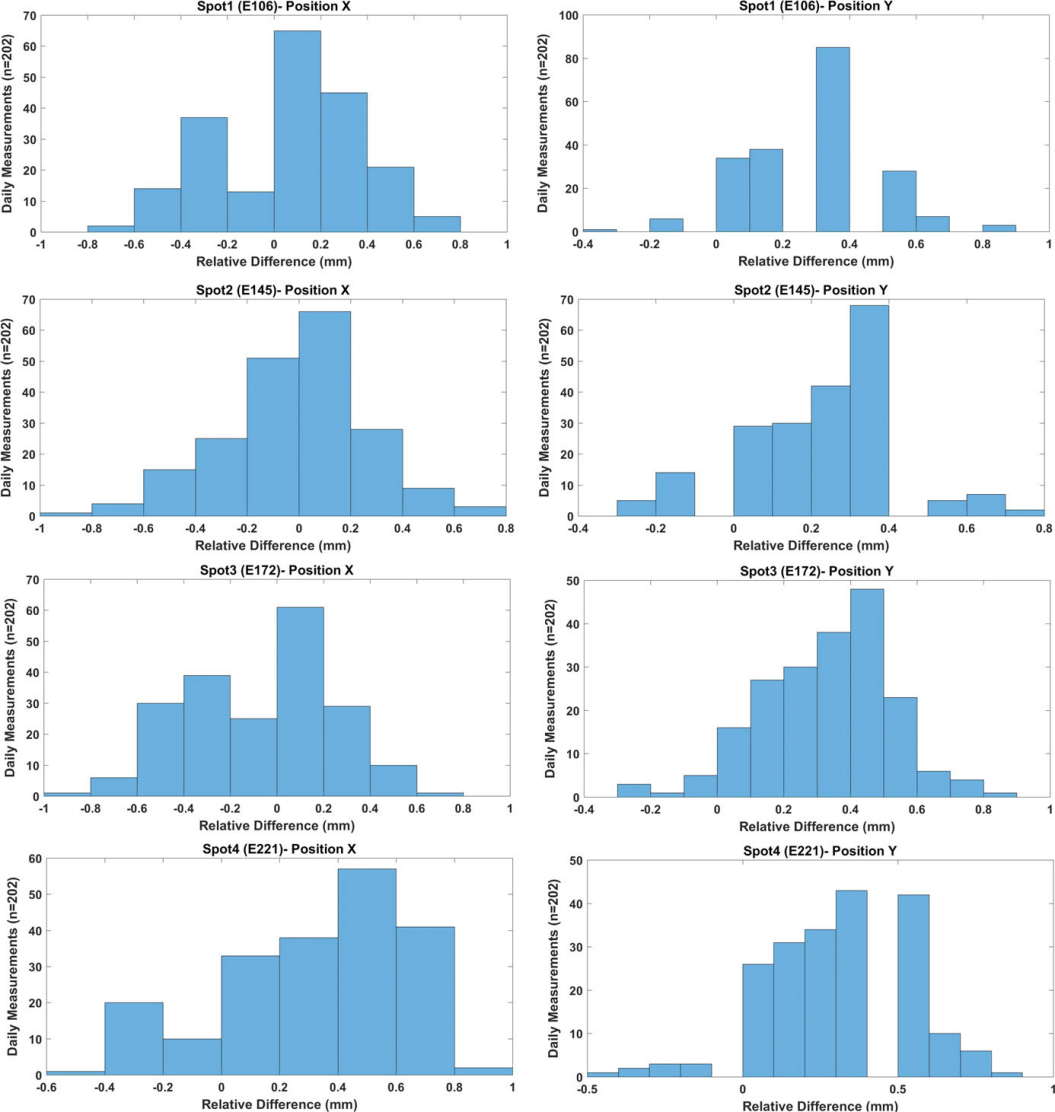
The difference (mm) in positions X and Y of spot1 (106 MeV), spot2 (145 MeV), spot3 (172 MeV), and spot4 (221 MeV). [The relative difference (Δ) was calculated by comparing daily (D) measurements (*n* = 202) against baseline (B) measurement.]

**Figure 12:**
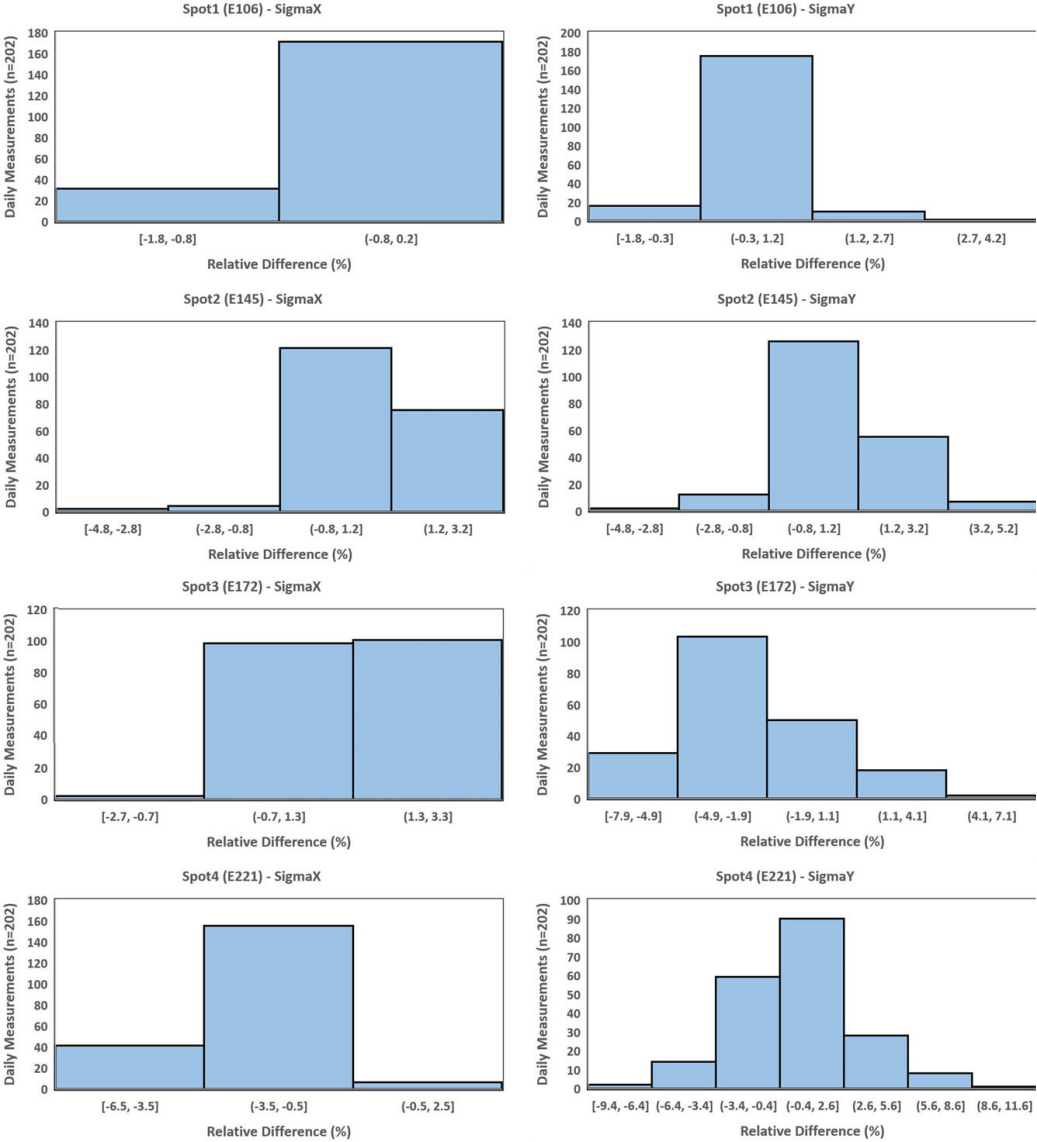
The difference (%) in Sigma X and Y of spot1 (106 MeV), spot2 (145 MeV), spot3 (172 MeV), and spot4 (221 MeV). [The relative difference (Δ) was calculated by comparing daily (D) measurements (*n* = 202) against baseline (B) measurement.]

Spots characteristics (position, size, and skewness) were evaluated for four spots (106, 145, 172, and 221 MeV). Table 3 and Figs. 11 and 12 show that the Δ in spots positions (X & Y) was within ±1 mm, and the Δ in in‐air spot sigma (X & Y) was within ±10% for all four spots. The 3*σ* (Table 3) evaluation of spot position showed that all four spots had slightly lower value in y direction (±0.4 mm) compared to the one in x direction (±0.6). For the in‐air spot sigma, the 3*σ* was found to increase with beam energy, and it increased from ±0.9% to ±2.1% for in‐air spot sigma X and from ±1.1% to ±3.6% for in‐air spot sigma Y. The Δ in spots skewness (X & Y) was within ±0.5 for all four spots (Table 3). The 3*σ* of spot skewness ranged from ±0.2 to ±0.3 (Table 3).

**Table 3:** Results of spots characteristics (position, sigma, and skewness) based on daily quality assurance measurements (*n* = 202)

	Avg.	Range	SPC		
			3*σ*	UCL	LCL
Spot position‐X (mm)					
Spot1 (E106)	0.0	−0.8–0.8	±0.6	0.6	−0.6
Spot2 (E145)	−0.1	−0.9–0.7	±0.6	0.5	−0.7
Spot3 (E172)	−0.1	−0.9–0.7	±0.6	0.6	−0.7
Spot4 (E221)	0.3	−0.5–0.9	±0.6	0.9	−0.3
Spot position‐Y (mm)					
Spot1 (E106)	0.3	−0.3–0.9	±0.4	0.7	−0.1
Spot2 (E145)	0.2	−0.3–0.7	±0.4	0.6	−0.2
Spot3 (E172)	0.3	−0.3–0.8	±0.4	0.7	−0.1
Spot4 (E221)	0.3	−0.4–0.9	±0.4	0.7	0.0
Spot sigma‐X (%)					
Spot1 (E106)	−0.3	−1.8–0.0	±0.9	0.6	−1.2
Spot2 (E145)	0.8	−4.8–2.4	±1.7	2.5	−1.1
Spot3 (E172)	1.3	−2.7–2.7	±1.9	3.2	−0.4
Spot4 (E221)	−3.8	−6.5–0.0	±2.1	−1.7	−5.9
Spot sigma‐Y (%)					
Spot1 (E106)	0.0	−1.8–3.6	±1.1	1.1	−1.1
Spot2 (E145)	0.6	−4.8–4.8	±2.0	2.6	−1.4
Spot3 (E172)	−1.9	−7.9–5.3	±2.7	0.9	−4.5
Spot4 (E221)	−0.7	−9.4–9.4	±3.6	2.8	−4.3
Spot skewness‐X					
Spot1 (E106)	0.1	−0.1–0.2	±0.2	0.3	−0.2
Spot2 (E145)	0.1	−0.1–0.3	±0.3	0.3	−0.2
Spot3 (E172)	0.0	−0.3–0.3	±0.3	0.3	−0.3
Spot4 (E221)	0.0	−0.3–0.3	±0.3	0.3	−0.3
Spot skewness‐Y					
Spot1 (E106)	0.0	−0.2–0.2	±0.2	0.2	−0.2
Spot2 (E145)	0.2	−0.1–0.5	±0.2	0.4	−0.1
Spot3 (E172)	−0.2	−0.4–0.0	±0.3	0.1	−0.5
Spot4 (E221)	0.0	−0.2–0.3	±0.3	0.3	−0.3

A relative difference (Δ) was calculated by comparing daily (D) measurements (*n* = 202) against baseline (B) measurements. Upper control limit (UCL) and lower control limit (LCL) are based on statics process control (SPC) charts. UCL = +3*σ* and LCL = −3*σ* are from the average value.

### 3.D X‐ray vs proton beam coincidence

**Figure 13:**
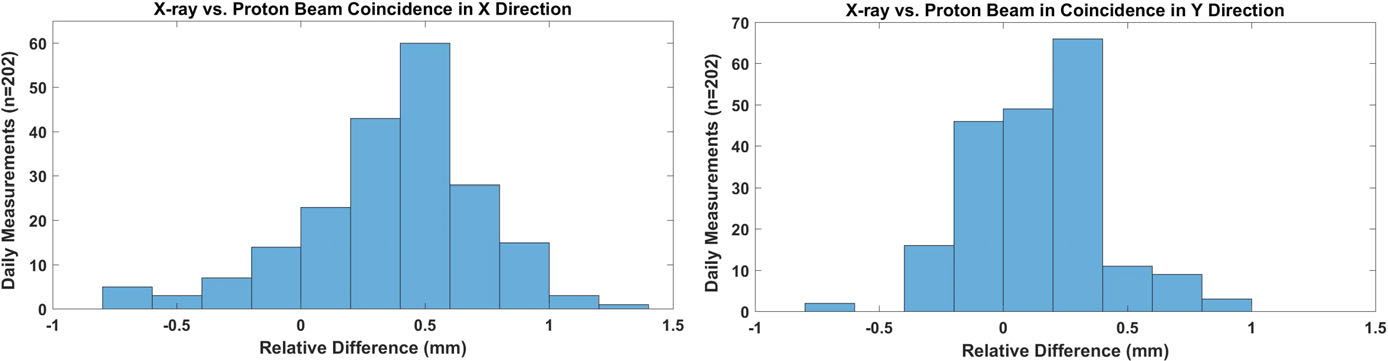
X‐ray vs proton beam coincidence in x and y directions. A single spot of 106 MeV was used for the coincidence.

Table 2 and Fig. 13 show that the Δ in x‐ray and proton beam coincidence (X and Y directions) was within ±1 mm except in one case (Δ = 1.3 mm). The 3*σ* of beam coincidence was ±0.7 mm in X and ±0.5 mm in Y directions (Table 2).

### 3.E Translational and rotational shifts

**Figure 14:**
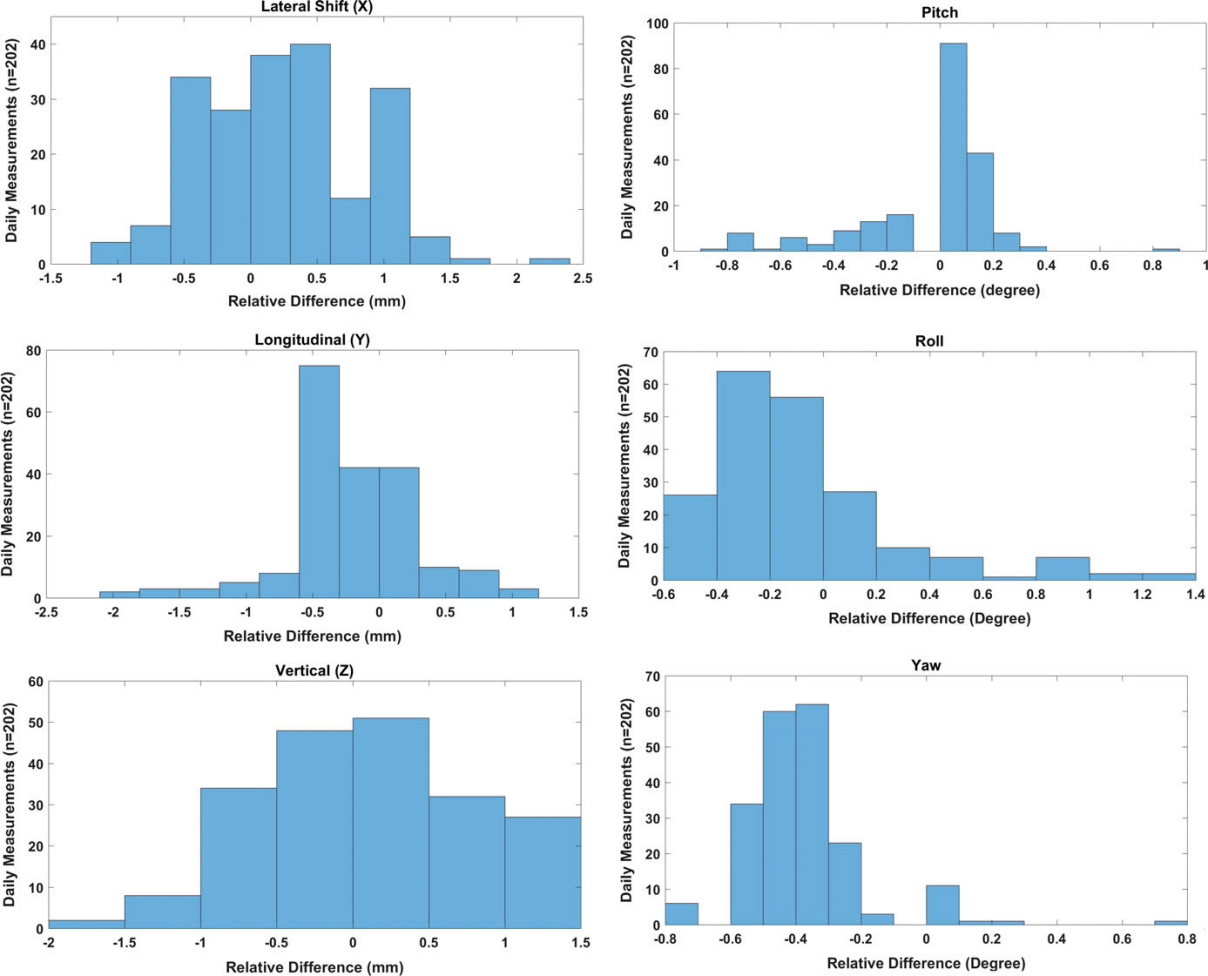
Difference between translational/rotational correction vectors and known offset (baseline) values for subsequent kV‐cone‐beam computed tomography imaging daily quality assurance measurements (*n* = 202).

Figure 14 shows the Δ in translational and rotational shifts from the baseline values. The Δ ranged from −1.0 to 2.3 mm in lateral, from −1.8 to 0.9 mm in longitudinal, and from −1.8 to 1.3 mm in vertical directions. For rotational shifts, the Δ ranged from −0.8° to 0.9° for pitch, from −0.6° to 1.4° for roll, and from −0.7° to 0.8° for yaw. The 3*σ* of the translational shifts was slightly higher in lateral (±0.8 mm) than in longitudinal (±0.6 mm) and vertical (±0.6 mm) directions, whereas for the rotational shifts, the 3*σ* was ±0.2° for yaw and ±0.3° for pitch and roll (Table 4).

**Table 4:** Comparison of 3*σ* values based on 202 sets of measurements with tolerance threshold from published literature

Daily QA tests	3*σ* based on control charts	Threshold from published literature
Dose output	±0.7%	±1%^8^, ±2%^5^, ^6^, ^9^, ±3%^4^
Field homogeneity	±0.5%	±2%^9^
Distal range	±0.3 mm	±0.5 mm^8^, ±1 mm^4^, ^5^, ±2 mm^6^, ^7^, ^9^
Proximal range	±0.3 mm	±2 mm^9^
Width	±0.2 mm	Not available
Distal‐fall‐off (mm)	0 mm for E106	Not available
	±0.1 mm for E145, E172 & E221	
Beam coincidence X	±0.7 mm	±1.5 mm^5^, ±2 mm^9^
Beam coincidence Y	±0.5 mm	
Spot position X	±0.6 mm	±1 mm^7^, ±1.5 mm^5^, ^8^, ±2 mm^6^, ^9^
Spot position Y	±0.4 mm	
In‐air spot sigma X	±0.9% for E106	±10%^5^, ^9^, ±15%^7^
	±1.7% for E145	
	±1.9% for E172	
	±2.1% for E221	
In‐air spot sigma Y	±1.1% for E106	
	±2.0% for E145	
	±2.7% for E172	
	±3.6% for E221	
Skewness X	±0.2 for E106	Not available
	±0.3 for E145, E172 & E221	
Skewness Y	±0.2 for E106 & E145	
	±0.3 for E172 & E221	
PPS translational	±0.8 mm for lateral (X)	±1 mm^4^, ^5^, ^8^, ^9^
	±0.6 mm for longitudinal (Y) and vertical (Z)	
PPS rotational	±0.3° for pitch and roll	Not available
	±0.2° for yaw	

## 4 DISCUSSION

The daily QA program discussed in this work was designed with a few concepts in mind, namely: (a) establish a comprehensive QA program incorporating proposed recommendations from forthcoming TG‐224,^9^ (b) mimic the typical patient treatment workflow to validate data transfer, and (c) utilize novel commercial devices to facilitate comprehensive and efficient testing. Due to the unique software infrastructure at our center, daily proton therapy treatments are delivered using multivendor hardware and associated software. Because of potential risks of connectivity issues, testing connectivity among the OIS and beam delivery/imaging systems on a daily basis helps validate the workflow functionality and can potentially detect issues prior to patient treatments. In developing a daily QA program for a PBS proton therapy system, there is currently limited guidance regarding the specific tests, frequency of testing, and tolerances for each test. Part of the challenge with standardized guidance stems from the variability in delivery technologies (e.g., gantry, fixed‐beam, etc.), techniques (e.g., double scatter, uniform scanning, etc.) as well as different IGRT imaging techniques (e.g., kV‐kV, CBCT, CT‐on‐rails, etc.). Currently, there is an AAPM TG‐224 working on a report to address these issues and provide recommendations; however, as of date, there is no official publication. There is, however, a growing consensus as to a limited set of tests and their accepted frequency from recent publications.^4^, ^5^, ^6^, ^7^, ^8^, ^9^ Although, it is important to mention that the determination of action level tolerances still remains a challenge.

Conceptionally, in attempting to establish tolerances for specific tests of a quality assurance program, a number of strategies may be employed to determine the tolerance action value. One such approach is to follow the recommended tolerances established by published guidelines that were conceived by the consensus of a group of experienced users — that is, for example, an AAPM task group report. A second could be to evaluate the impact on the patient dose distribution due to variations in that specific parameter. For example, there have been publications characterizing the impact of spot size on treatment plan quality.^15^ A third approach is to use statistical process control to evaluate whether specific parameters are behaving in a stable and controlled manner.^16^, ^17^ With this information, it is possible to use statistical methods to determine the system specific action level tolerances due to the system performance. In using statistical process control, the methodology is to first establish a process of testing a parameter, test and observe, and characterize the behavior of the specific parameter — for example, dose output, spot size, spot position, etc. — over a time period. By characterizing the behavior, it is possible to determine when a parameter is out of control and is statistically an outlier. This helps provide guidance as to when to act. In this study, 10 months’ worth of data was collected to characterize the behavior of our proton PBS delivery system. Our goal was to measure the stability of multiple parameters and establish tolerances based on our specific system performance and not on generic guidelines. With the assumption that a parameter value is approximately distributed normally, control limits based purely on the behavior of the variability can be generated. Using control charts, a delivery system‐specific action level (3*σ*) table can be determined for daily QA.

With regards to dose output, the AAPM TG 142^18^ recommends the tolerance of 3% for photon and electron, whereas Lambert et al.^5^ and Actis et al.^6^ have used a tolerance of 2% and Younkin et al.^8^ used tolerance of 1% for PBS protons. In our current daily QA setup, dose output is typically measured by taking at least two readings with a PPC05 chamber. The statistical process control analysis results from the past 10 months show that the 3*σ* of dose output is ±0.7%, which is a tighter tolerance compared to the published literature.^4^, ^5^, ^6^, ^8^, ^9^ For proton range and energy, authors have used different tolerances of 0.5 mm,^8^ 1 mm,^4^, ^5^ and 2 mm.^6^, ^7^, ^9^ For our 10‐month data, we noticed the variation in range (R80) within ±0.5 mm for all four energies evaluated (106, 145, 172, and 221 MeV). The 3*σ* of daily range tolerance of ±0.3 mm is reasonable on ProteusPLUS PBS proton machine if the institution uses the Sphinx and myQA software for daily range verification.

Looking at proton beam characteristics such as in‐air spot size, Lambert et al.^5^ and Bizzochi et al.^7^ evaluated in‐air spot size of a single energy with tolerances of ±10% and ±15%, respectively. However, if the institution evaluates multiple spots of different energies on a daily basis, a single tighter tolerance value for all energies may not be ideal. For spots characteristics, we deliver four spots of different energies (106, 145, 172, and 221 MeV) and myQA software is used to analyze the in‐air spot size (sigma). For a spot of 221 MeV, our daily measured in‐air spot sigma deviated from the baseline value by up to ±9.5%, whereas for the lower energy spots (106 and 145 MeV), the deviation of daily in‐air spot sigma from baseline value was <±5%. The 3*σ* of in‐air spot size also showed a similar trend such that there is an increase in deviation with energy. Additionally, there can be difference in X and Y directions of in‐air spot size, especially at higher energies, on an IBA ProteusPLUS proton therapy system. For spot position tolerance, there is no common agreement among investigators. For instance, spot position tolerance of ±1 mm from Bizzochi et al.^7^ is more stringent than ±1.5 mm suggested by Lambert et al.^5^ and Younkin et al.^8^ and ±2 mm applied by Actis et al.^6^ Although our daily spot positions (a total of four spots with energies 106, 145, 172, and 221 MeV) varied from baselines values by up to ±0.9 mm on certain days, the 3*σ* of spot positions was lower (±0.6 mm in X and ±0.4 mm in Y). Since the Sphinx and Lynx are indexed on the couch top, the accuracy of robotic couch could also potentially affect the spot positions results. Hence, the verification of daily QA setup with planar kV x rays is essential to reduce the uncertainties introduced by the user setup and robotic couch. For our current daily QA protocol and workflow (Fig. 6), a tighter spot position tolerance of ±0.6 mm is feasible.

For x‐ray and proton beam coincidence, we currently use a single spot of energy 106 MeV. Based on 10 months results, the deviation in coincidence was found to be within ±1.5 mm, which was used as the tolerance by Lambert et al.^5^ The 3*σ* of beam coincidence (x ray and proton) was found to be ±0.7 mm in X and ±0.5 mm in Y directions. As shown in Fig. 6, we use the setup field to drive the 6D robotic couch to its predefined position such that the fiducial (2 mm in diameter) that is a part of our indexed couch top setup is at the imaging isocenter. Once the portal kV‐kV x‐ray image of the setup is acquired, the center of cross‐hair (imaging isocenter) is projected at the center of fiducial manually. It was found that the combination of accuracy of robotic couch, phantom setup, and manual alignment of cross hair at the center of fiducial could affect the localization of the fiducial at the imaging isocenter. Hence, the robust indexing of the phantom along with well‐defined manual alignment process of the fiducial with imaging isocenter is critical in determining the coincidence of x‐ray and proton beam.

In our current patient treatment workflow, we typically acquire CBCT images followed by orthogonal kV‐kV x‐ray images. Although the use of Sphinx and Lynx for daily imaging QA would be more effective in reducing total daily QA time in the treatment room, we noticed that the CBCT acquisition and automatic image registration in adaPT Insight for Sphinx and Lynx is not optimal. Hence, the MIMI phantom in conjunction with the HexaCheck is used to assess the 6D correction vector, which is calculated based on the automatic rigid registration of the acquired CBCT images to the reference CT images of the MIMI Phantom. Lambert et al.^5^ and Younkin et al.^8^ have provided ±1 mm as the tolerance of couch correction vector. Both of these publications^5^, ^8^ utilized the kV‐kV x‐ray imaging of the DQA‐3 device to calculate the correction vector for translational shifts only, whereas we have utilized the CBCT of the MIMI/HexaCheck to assess the 6D correction vector, which includes both the translational and rotational shifts. Based on 202 sets of measurements, the 3*σ* of translational shifts (lateral, longitudinal, and vertical) ranged from ±0.6 to ±0.8 mm, and the 3*σ* of rotational shifts (pitch, roll, and yaw) ranged from ±0.2° to ±0.3°. The variation in daily 6D correction vector in our current daily QA setup is found to be mainly due to the combination of (a) reproducibility of MIMI/HexaCheck setup on the couch top, (b) accuracy of 6D LEONI robotic couch, (c) user dependency on selection of region of interest (ROI) for image registration in adaPT Insight, and (d) image registration algorithm implemented within adaPT Insight imaging system.

In addition to planar kV x rays and CBCT, QA on the C‐RAD CatalystHD surface imaging is performed daily by utilizing a vendor supplied daily QA phantom. Following the TG‐147 daily QA recommendations, the functionality of the CatalystHD system and coincidence of the surface imaging and laser isocenter is verified. Specifically, the C‐RAD daily QA phantom is aligned to the room isocenter using the room/gantry lasers. Once positioned, the daily QA phantom is imaged, and the agreement between the laser isocenter and surface imaging isocenter is quantified. Tolerances and stability have been previously reported by Stanley et al.^19^ Our current surface imaging daily QA tests include the laser accuracy, functionality of the system, and calculation of translational shifts (tolerance ±1 mm).

Lastly, for many proton centers, efficiency is an important element being that beam access is limited. Recently, published literature have reported proton daily QA time of 10 min,^8^ 20 min,^7^ and 30 min.^5^, ^6^ At our center, the daily QA time is under 30 min, which also includes the workflows presented in Fig. 6 and 8. The variation of daily QA time among different studies is mainly due to an inconsistency in the number and type of daily QA tests being performed at different institutions. For example, the coincidence of x‐ray and proton beam is tested by Lambert et al.^5^ only, and in‐air spot size is reported by Lambert et al.,^5^ Actis et al.,^6^ and Bizzochi et al.^7^ Furthermore, none of the studies^4^, ^5^, ^6^, ^7^, ^8^ reported the CBCT acquisition and its functionality as a part of daily QA. This could be due to unavailability of CBCT in the treatment room or difference in daily QA policies at the authors’ institutions.^4^, ^5^, ^6^, ^7^, ^8^ The inclusion of CBCT in our daily QA workflow (Fig. 8) has certainly contributed about 5 min toward the total daily QA time at our center. In addition of calculating the 6D correction vectors by using CBCT, this test allows us to test the functionality of x‐ray tube, collision detection software, and adaPT Insight as well as transfer of CBCT images to the OIS for offline review.

## 5 CONCLUSION

With the increasing complexity of delivery, patient positioning, and imaging systems, a robust and comprehensive daily QA program is required to gain confidence in the performance of a proton therapy system. The use of novel phantoms and dosimetry devices such as the Sphinx in conjunction with the Lynx and HexaCheck/MIMI was shown to provide a robust, consistent and efficient method of evaluating various aspects of our delivery system which include PBS beam parameters and imaging/couch accuracy. Our daily QA results from over 10 months demonstrate consistent beam stability of the ProteusPLUS PBS proton therapy system. If CBCT is available, it is recommended to test its functionality on a daily basis mimicking a patient treatment scenario. The use of MIMI/HexaCheck can serve an accurate and efficient tool to perform daily, 6D IGRT QA of the IBA adaPT Insight software and LEONI robotic couch.

## CONFLICT OF INTEREST

The authors declare no conflict of interest.
